# Transient Global Amnesia Associated with a Unilateral Infarction of the Fornix: Case Report and Review of the Literature

**DOI:** 10.3389/fneur.2014.00291

**Published:** 2015-01-12

**Authors:** Mihir Gupta, Molly A. Kantor, Christie E. Tung, Niushen Zhang, Gregory W. Albers

**Affiliations:** ^1^Stanford University School of Medicine, Stanford, CA, USA; ^2^Department of Internal Medicine, Stanford Hospital and Clinics, Stanford, CA, USA; ^3^Department of Neurology, Stanford Hospital and Clinics, Stanford, CA, USA; ^4^Stanford Stroke Center, Stanford University, Stanford, CA, USA

**Keywords:** amnesia, transient global, brain infarction, fornix, brain, stroke

## Abstract

Stroke is an extremely uncommon cause of transient global amnesia (TGA). Unilateral lesions of the fornix rarely cause amnesia and have not previously been reported to be associated with the distinctive amnesic picture of TGA. We describe the case of a 60-year-old woman who presented with acute onset, recent retrograde, and anterograde amnesia characteristic of TGA. Serial magnetic resonance imaging showed a persistent focal infarction of the body and left column of the fornix, without acute lesions in the hippocampus or other structures. Amnesia resolved in 6 h. Infarction of the fornix should thus be included in the differential diagnosis of TGA, as it changes the management of this otherwise self-limited syndrome.

## Introduction

Transient global amnesia (TGA) is a clinical syndrome characterized by transient loss of recent retrograde and anterograde memory with preservation of immediate recall, remote memory, visual-spatial function, attention, and language (1). Most patients are over age 50, and rarely exhibit other neurological deficits. Symptoms resolve within 24 h and recurrence is rare. Although the etiology is incompletely understood, TGA may be related to vulnerability of hippocampal CA1 neurons to metabolic stress. Focal 1–5 mm lesions in this region are seen on high-resolution magnetic resonance imaging (MRI) in up to 85% of cases; these lesions are most often detected 24–72 h after clinical onset and persist for 7–10 days, but eventually resolve (2). Arterial ischemia and migraine are not generally considered causes of TGA, but rare cases have been associated with ischemic lesions in the hippocampus, splenium of the corpus callosum, or thalamus (1). To our knowledge, lesions in other structures involved in memory, including the fornix, have not previously been associated with TGA.

The fornix comprises efferent fibers from the hippocampus to the mammillary bodies and anterior thalamic nuclei, connecting structures involved in processing and storing memory. While amnesia may occur following ischemia of the bilateral fornices (3), isolated unilateral lesions of the fornix rarely cause amnesia. Because many patients with fornix lesions do not develop memory deficits, the extent to which integrity of the fornix is essential for memory is unclear. When amnesia does occur, severe and persistent impairment in delayed recall is a characteristic feature; these patients have not been reported to develop recent retrograde and anterograde amnesia characteristic of TGA. We report the first case, to our knowledge, of an isolated unilateral infarction of the fornix in a patient presenting with TGA.

## Case Report

A 60-year-old woman presented to the Emergency Department at our institution with sudden onset of retrograde amnesia for events of the previous day along with severe anterograde amnesia. Although she had spent the day doing various chores with her brother, she could not recall any events after eating breakfast. She repeatedly asked her brother the date and what they had done that day, despite just having heard the answer. She was unable to recall other recent events such as a family friend falling ill or political news she had been following. Her symptoms began while running errands, approximately 3–5 h after undergoing an abdominal CT scan. She could not identify any other discrete stressful event preceding symptom onset. She endorsed intermittent headaches for the previous 5 days that lasted about 45 min without features of migraine; some worsening of long-standing mild left-sided residual weakness from prior embolic stroke; and a recent urinary tract infection.

Past history was notable for paroxysmal atrial fibrillation complicated by prior embolic stroke and retinal artery occlusion, bicuspid aortic valve status-post prosthetic aortic valve replacement, hypertrophic cardiomyopathy, prior non-ST elevation myocardial infarction (NSTEMI) secondary to demand ischemia, cervical myelopathy, hypothyroidism, hyperlipidemia, and depression. She never used tobacco, alcohol, or illicit drugs. Medications included warfarin, celecoxib, dronedarone, ezetimibe, colesevelam, furosemide, spironolactone, levothyroxine, and zolpidem.

Physical examination was notable for mild hypertension (136/99 mm Hg) and a grade III/VI harsh crescendo-decrescendo systolic murmur at the left upper sternal border. She was alert and oriented, without facial droop, dysarthria, aphasia, diplopia, or changes in hearing, taste, or sensation. She had normal cranial nerves, sensory and cerebellar function, narrow-based shuffling gait, brisk and symmetric reflexes, equivocal Babinski’s sign, and left pronator drift. Power was 4/5 in the left upper and lower extremity, as well as the right wrist extensors, finger extensors, and hallucis longus. Laboratory tests were remarkable for mild leukocytosis with neutrophilia, therapeutic INR (2.5), microscopic hematuria, and normal electrolytes and renal and liver function. Troponin was mildly elevated (0.7 ng/mL) and EKG showed no ST elevation. Head CT without IV contrast showed scattered intraparenchymal calcifications consistent with old neurocysticercosis, and no acute abnormalities.

**Figure 1:**
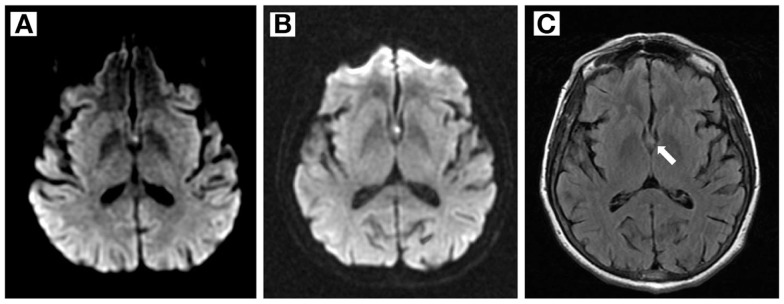
**Magnetic resonance imaging findings**. **(A,B)** Diffusion- weighted imaging shows a small hyperintense area in the left fornix 1 and 3 days after symptom onset, respectively. **(C)** FLAIR image 3 days after symptom onset.

Aspirin 81 mg and atorvastatin 80 mg were administered in the Emergency Department based on initial suspicion for nSTEMI or stroke. Amnesic symptoms resolved within 6 h of onset. The mild pronator drift, shuffling gait, and exacerbation of left-sided weakness also resolved. MRI and MRA with Multihance IV contrast on a 1.5 T scanner showed a small focus of restricted diffusion involving the body and left column of the fornix consistent with acute infarction (Figure 1), normal cerebral perfusion without flow-limiting stenosis, and scattered old calcifications also seen on CT. Repeat MRI 3 days after symptom onset again demonstrated acute infarction of the left fornix; no new lesions were seen. Sequences included axial DWI, GRE and FLAIR, ASL, time-of-flight, and bolus perfusion.

At 1-month follow-up evaluation, the patient was asymptomatic. Neurological examination was normal. Folstein mini-mental status examination score was 30/30 on the day following initial presentation (approximately 12 h after TGA symptoms had resolved), over the next 2 days of inpatient stay and at 1-month follow-up.

## Discussion

Our patient presented with classic symptoms of TGA and had evidence of unilateral infarction of the left fornix on imaging. To our knowledge, there is only one previous report of an isolated unilateral infarction of the fornix (4). That patient presented with a sudden onset of both anterograde and retrograde amnesia, slowing of processing speed, and sparing of short-term memory. MRI revealed an isolated left fornix infarction. On early follow-up, severe reduction in delayed recall (classical of fornix lesions) and reduced verbal, visual, and general memory persisted. Notably, the occurrence of impairment of visual memory raises the possibility of at least some damage to the right fornix or other structures that was not visible on imaging. In contrast, our patient had no clinical or radiological features to suggest involvement of any other structures, and the clinical presentation was typical of TGA rather than the amnesic syndrome associated with fornix lesions.

Previous reports have described memory impairment associated with unilateral lesions of the fornix, usually a consequence of accidental trauma or surgical injury rather than infarction (Table 1) (5–10). Lesions were often caused by sectioning of or injury to one side of the fornix in order to gain access to the foramen of Monro and the third ventricle. Most patients had damage to additional structures, making it difficult to definitively attribute the resulting neurological dysfunction to the fornix lesion itself. Other cases (not reviewed here) had bilateral damage to the fornix, which appears sufficient to induce amnesia.

**Table 1:** **Previously reported cases of unilateral fornix lesions**.

Patient	Etiology	Fornix lesion	Other lesions	Symptoms	Outcome and follow-up	Other	Reference
52 years M	Infarction	Left fornix anterior column	None	Sudden onset anterograde and retrograde amnesia, reduced processing speed.	1 month: impaired delayed recall, and verbal, visual and general memory. 3 months: memory impairment persisted	MRI at 4 months showed a small persistent cavity in the left fornix	Korematsu et al. (4)
47 years M	Surgical resection of orbitofrontal pilocytic astrocytoma	Right fornix	Right orbitofrontal gyrus, anterior cingulate cortex, inferior and middle frontal gyrus, right frontal lobe corona radiata, right hypothalamus	At 3 months, impaired retrograde memory, conceptual reasoning, flexibility, long-term verbal and spatial memory, delayed recall. Severe confabulation	17 months: continued impairment of delayed recall verbal memory. Normalized executive functions, confabulation processing speed and memory.	Symptom attribution: confabulation (orbitofrontal lesion), amnesia (right fornix, commisure of fornix and cingulate cortex), delayed recall deficit (right hemisphere)	Ruggeri and Sabatini (9)
18 years M	TBI, DAI, occipital SAH	Right fornix crus (3 months), bilateral fornix (14 months)	Degeneration both ends of left cingulum. Encephalomalactic lesion in posterior corpus callosum at 3 months	LOC for 7 days. At 3 months, mild right hemiparesis, cognitive impairment (IQ 75), marked memory impairment	14 months: weakness resolved, IQ improved (105), memory worsened.	Evaluated by MRI and DTI/DTT at 3 and 14 months	Hong and Jang (6)
28 years M	TBI, DIA, left temporal ICH	Left fornix crus	Focal leukomalactic lesion in corpus callosum.	At 3 months, impaired verbal memory (18th percentile), spared visual memory (81st percentile)	N/A	Old hemorrhage in left temporal lobe and pons. Evaluated by MRI and DTI/DTT at 3 months	Hong and Jang (6)
55 years F	Surgical resection of third ventricle cyst	Right fornix and posteriorly	Right prefrontal cortex tissue and white matter, posterior right thalamus. Hippocampus and mammillary bodies degenerative changes.	Initially asymptomatic	9 years: delayed visuo-spatial memory. Mild impairment in a difficult delayed verbal memory test.	Post-operative right VP shunt. Symptom attribution: visuo-spatial memory (right fornix), verbal memory (frontal lobe)	McMackin et al. (8)
25 years F	Surgical resection of malignant astrocytoma	Left fornix	Tumor in left fornix, posterior thalamus, hippocampus, left lateral ventricle and through ventricular wall	Impairment of recent memory	3 months: severe impairment in immediate and delayed verbal memory.	Adjuvant radiation. Post-operative MRI: residual tumor through ventricular wall, transecting left fornix crus, posterior thalamus and hippocampus	Tucker et al. (10)
N/A	Surgical resection	Right fornix	N/A	Memory impairment	Memory impairment persisted on follow-up	Resection of glioma (first pt) and colloid cyst (second pt)	Carmel (5)

*DAI, diffuse axonal injury; DTI/DTT, diffusion tensor imaging/tractography; ICH, intracerebral hemorrhage; LOC, loss of consciousness; MRI, magnetic resonance imaging; N/A, not available or unspecified; SAH, subarachnoid hemorrhage; TBI, traumatic brain injury; VP, ventriculo-peritoneal shunt*.

Similarly, while cases of infarction of the fornix resulting in memory loss have been reported, these patients had bilateral fornix ischemia or simultaneous ischemic damage to additional structures involved in memory, making it difficult to attribute the amnesia to the fornix lesion alone (3). When they do cause symptoms, unilateral lesions of the right fornix typically cause impairment of visual-spatial memory, whereas lesions of the left fornix result in verbal memory dysfunction, possibly due to the laterality of hippocampal function. Consistent with this notion, hippocampal lesions do not cause global amnesia, but rather impair learning of specific tasks. Our patient had none of the previously reported symptoms of left fornix infarction.

Instead, our patient presented with a classic case of TGA. However, the characteristic pattern of memory loss as well as the etiopathological and anatomical correlates in TGA (1) are quite distinct from those previously reported in patients with lesions of the fornix. Although most patients with TGA have focal lesions in the CA1 region of the hippocampus detected 24–72 h after symptom onset (2), these were absent in our patient on serial MRI/DWI scans obtained 1 and 3 days after symptom onset. We, therefore, exclude the possibility that hippocampal lesions were present and caused the clinical manifestations of TGA. There was no evidence of involvement of other regions of the brain. The transient worsening of left-sided weakness likely represented unmasking of her long-standing mild left-sided weakness, since no new lesions were seen in the corresponding regions on repeated imaging.

Our patient did not participate in any physical activities, or have any of the clinical or psychological events, medical procedures, or medication changes reported to precipitate TGA. She had numerous risk factors for cardioembolic and atherosclerotic small vessel occlusive stroke, as well as for intracranial hemorrhage given concurrent use of both warfarin and celecoxib. This infarction was unlikely due to cardioembolism, despite the history of atrial fibrillation because the vascular supply of the fornix consists of small perforating branches of the anterior communicating or proximal anterior cerebral arteries. Our patient was treated with standard secondary stroke risk factor modification, including continuing her anticoagulation with warfarin, a cholesterol-lowering agent, blood pressure recommendations, and lifestyle coaching.

## Conclusion

Transient global amnesia can occur in association with small, isolated unilateral lesions of the fornix, extending the locations of lesions that cause this syndrome. Since TGA is rarely associated with irreversible brain lesions, resolves spontaneously, and usually does not recur, standard management is expectant. Timely evaluation by MRI should be strongly considered in cases of TGA in order to exclude the possibility of infarction, particularly in patients with risk factors for stroke. Though a rare cause of TGA, infarction should be included in the differential diagnosis since it will change management and prognosis.

## Author Contributions

*Study concept and design:* Mihir Gupta, Molly A. Kantor, Christie E. Tung, and Gregory W. Albers. *Acquisition, analysis, or interpretation of data:* Mihir Gupta, Molly A. Kantor, Christie E. Tung, and Gregory W. Albers. *Drafting of the manuscript:* Mihir Gupta, Molly A. Kantor, Christie E. Tung, Niushen Zhang, and Gregory W. Albers. *Critical revision of the manuscript for important intellectual content:* Mihir Gupta, Molly A. Kantor, Christie E. Tung, Niushen Zhang, and Gregory W. Albers. *Study supervision:* Gregory W. Albers.

## Conflict of Interest Statement

The authors declare that the research was conducted in the absence of any commercial or financial relationships that could be construed as a potential conflict of interest.
